# Evaluation of Mortality in Atrial Fibrillation: Clinical Outcomes in Digital Electrocardiography (CODE) Study

**DOI:** 10.5334/gh.772

**Published:** 2020-07-28

**Authors:** Gabriela M. M. Paixão, Luis Gustavo S. Silva, Paulo R. Gomes, Emilly M. Lima, Milton P. F. Ferreira, Derick M. Oliveira, Manoel H. Ribeiro, Antonio H. Ribeiro, Jamil S. Nascimento, Jéssica A. Canazart, Leonardo B. Ribeiro, Emelia J. Benjamin, Peter W. Macfarlane, Milena S. Marcolino, Antonio L. Ribeiro

**Affiliations:** 1 Telehealth Network of Minas Gerais, Hospital das Clínicas and Faculdade de Medicina, Universidade Federal de Minas Gerais, Belo Horizonte, Minas Gerais, BR; 2 Department of Medicine, Boston University School of Medicine, Department of Public Health, Boston University School of Public Health, Boston, MA, US; 3 Division of Cardiovascular and Medical Sciences, University of Glasgow, UK

**Keywords:** electronic cohort, electrocardiogram, atrial fibrillation, mortality

## Abstract

**Aims:**

Atrial fibrillation (AF) is a public health problem and its prevalence is increasing worldwide. Electronic cohorts, with large electrocardiogram (ECG) databases linked to mortality data, can be useful in determining prognostic value of ECG abnormalities. Our aim is to evaluate the risk of mortality in patients with AF from Brazil.

**Methods:**

This observational retrospective study of primary care patients was developed with the digital ECG database from the Telehealth Network of Minas Gerais, Brazil. ECGs performed from 2010 to 2017 were interpreted by cardiologists and the University of Glasgow automated analysis software. An electronic cohort was obtained linking data from ECG exams and those from a national mortality information system, using standard probabilistic linkage methods. We considered only the first ECG of each patient. Patients under 16 years were excluded. Hazard ratios (HR) for mortality were adjusted for demographic and self-reported clinical factors and estimated with Cox regression.

**Results:**

From a dataset of 1,773,689 patients, 1,558,421 were included, mean age 51.6 years; 40.2% male. There were 3.34% deaths from all causes in 3.68 years of median follow up. The prevalence of AF was 1.33%. AF was an independent risk factor for all-cause mortality (HR 2.10, 95%CI 2.03–2.17) and cardiovascular mortality (HR 2.06, 95%CI 1.86–2.29). Females with AF had a higher risk of overall and cardiovascular mortality compared with males (p < 0.001).

**Conclusions:**

AF was a strong predictor of cardiovascular and all-cause mortality in a primary care population, with increased risk in women.

**Condensed abstract:**

To assess risk of mortality in AF patients, an electronic cohort was obtained linking data from ECG exams of Brazilian primary care patients and a national mortality information system. From 1,558,421 patients, AF (prevalence 1.33%) carried a higher risk of overall and cardiovascular mortality, with increased risk in women.

**What’s New:**

- This is the first study with a large Brazilian electronic cohort to evaluate the risk of mortality linked to AF in primary care patients.
- AF patients from a Brazilian primary care population had a higher risk of death for all causes (HR 2.10, 95%CI 2.03–2.17) and cardiovascular mortality (HR 2.06, 95%CI 1.86–2.29).
- Female patients with AF had an increased risk of overall and cardiovascular mortality compared with male patients (p < 0.001).

## Introduction

As the world population ages, the prevalence of atrial fibrillation (AF) is increasing, with a consequent increase in medical and economic global burden [1]. It is estimated that about 0.5 to 1% of the general population has AF and the lifetime risk of AF is about one in three in white and one in five in black middle-aged individuals in Europe and United States [2,3]. Despite the higher prevalence in high-income countries, low- and middle-income countries also have witnessed a progressive increase in their AF prevalence [1]. In a previous study from our group, in a Brazilian primary care population, the overall AF and atrial flutter prevalence was 1.8% [4].

Atrial fibrillation increases the risk of all-cause mortality, cardiovascular mortality and morbidity [5,6]. Stroke in AF patients is usually more severe with worse prognosis and higher costs to the health system [7]. Costs related to AF are responsible for 1% of the National Health Service budget in the United Kingdom and, in the United States, overall AF hospitalizations increased by 23% from 2000 to 2010, with an increase of 24% of the mean cost of hospitalizations, without significant change in the mean length of stay [8,9]. Half of the world population is concentrated in a few rapidly developing countries: Bangladesh, Brazil, China, India, Indonesia, Nigeria, and Pakistan [10]. In these countries, populations of individuals over 60 years are predicted to at least double by 2050 [10]. Worryingly, with rapidly ageing populations, the costs of AF are especially likely to balloon [10].

There are few studies on AF mortality and its risk factors in low- and middle-income countries, especially in a primary care population. In Brazil, AF and atrial flutter mortality rate was estimated in 2.8 per 100,000 inhabitants, standardized by age [11]. The use of electronic cohorts, with large electrocardiogram (ECG) databases linked to mortality data, can be an important tool in determining the prognostic value, in a real setting, of established and new ECG markers. Our aim is to evaluate the mortality in AF patients of a large electronic cohort.

## Methods

This is an observational retrospective study, developed using the database of digital ECGs from the Telehealth Network of Minas Gerais (TNMG) [12], which is responsible for more than 800 municipalities in a public Brazilian telehealth system. It has acquired more than 4 million ECGs since its inception in 2006.

Minas Gerais is a state in southeast Brazil with a large number of municipalities (853), most of them small, and more than 20 million inhabitants. It can be considered representative of the country [13], as age distribution and percentage of urbanization are similar to the overall national pattern, as is the social inequality: the north and the northeast of Minas Gerais have a low Human Development Index (HDI) similar to the North and Northeast Brazil, while the west and south of the state have an HDI similar to the areas with the highest HDI of the country [14]. The HDI was published by the United Nations Development Program as a composite statistic of life expectancy index, education index, and income index used to rank countries or cities [14].

All ECGs recorded by the TNMG from patients at least 16 years-old from 2010 to 2017 were assessed. Exams without valid tracings or with technical problems were excluded. In patients who underwent more than one ECG, only the first exam was analyzed. ECGs were performed by the local primary care professional, using digital electrocardiographs by *Tecnologia Eletrônica Brasileira* model ECGPC (São Paulo, Brazil) or *Micromed Biotecnologia* model *ErgoPC 13* (Brasília, Brazil).

Clinical data (age, sex, and comorbidities) were self-reported and collected using a standardized questionnaire by health professionals at the primary care unit at the same time of the ECG exam. Clinical conditions included self-reported smoking, hypertension, diabetes, dyslipidemia, Chagas disease, previous myocardial infarction and chronic obstructive pulmonary disease (COPD),

Specific software was developed in-house, capable of capturing an ECG tracing for immediate upload, along with the patient’s self-declared clinical history, to the TNMG analysis center via the internet. The clinical information, ECGs tracings and reports were stored in a customized database. For the purpose of the present study, the Glasgow University Interpreter program, an automatic 12 lead ECG analysis software, (release 28.4.1, issued on June 16th 2009) was used to automatically interpret all ECGs available in the database, exporting the diagnoses, accompanied by the corresponding Minnesota codes [15]. The Minnesota Code was created in 1960 for standardizing the electrocardiograph description and has become the method of choice for electrocardiography in epidemiological studies.

ECGs were also interpreted by a team of trained cardiologists using standardized criteria, in order to generate an ECG report, which was done as free text. A hierarchical free-text machine learning tool was used to recognize a diagnosis of AF among these reports. First, the text was preprocessed by removing stop-words and generating n-grams. Then, we used the Lazy Associative Classifier [16,17,18], a machine learning automatic classification software, which was built with a 2800-sample dictionary, manually created by specialists, based on text from real diagnoses. The final report was obtained by imputing the Lazy Associative Classifier results to a decision tree for class disambiguation. The decision tree was trained using the original dataset. The classification model was tested on 4557 medical reports manually labeled with 99% accuracy, 100% positive predictive value and 99% sensitivity.

**Figure 1:**
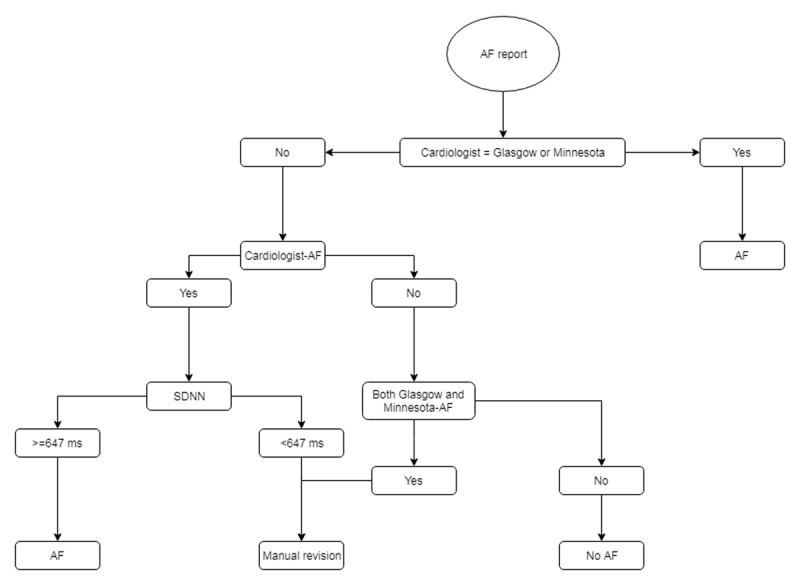
Diagram for atrial fibrillation diagnosis in the ECG database.

The electrocardiographic diagnosis of AF was accepted automatically when there was agreement between a cardiologist report and the automatic report from the Glasgow program or the Minnesota Code. In cases where there were discordances between the medical report and one of the automatic programs, some heuristic rules such as Standard Deviation of Normal to Normal RR intervals (SDNN) ≥647 ms (ROC curve area = 0.94) were used to automatically identify AF. If SDNN <647 ms, a manual revision was done in 4343 exams by trained staff. Those for which AF was diagnosed only by one of the automatic systems were not considered as AF (Figure 1). Atrial flutter was not considered as AF in this study.

The electronic cohort was obtained by linking data from the ECG exams (name, sex, date of birth, city of residence) and those from the national mortality information system, using standard probabilistic linkage methods (FRIL: Fine- grained record linkage software, v.2.1.5, Atlanta, GA).

The primary endpoint was all-cause mortality. The secondary endpoint was cardiovascular mortality, defined by cardiovascular disease through the International Classification of Diseases (ICD) coding: I00-I99, G45-G46.8, A39.52, B33.2-B33.24, D86.85 and A39.51.

R program (version 3.4.3, Vienna, Austria) was used for statistical analysis. Categorical data were reported as counts and percentages while continuous variables were reported as mean and SD or median (25th, 75th percentiles), as appropriate. The time elapsed between the date of the electrocardiogram (index event) and the event of interest (date of death) was considered a dependent variable. The presence of the AF was an independent variable, along with the clinical characteristics of the population. Patients who did not present the event of interest until the end of the follow-up were censored, but contributed with follow-up time until the end of the study (September 2017).

To assess the relation between AF and mortality, we analyzed Cox proportional regression, reporting hazard ratios and 95% confidence intervals, adjusted for age, sex, and clinical conditions (hypertension, diabetes, dyslipidemia, previous myocardial infarction, smoking, COPD, and Chagas disease). A two-tailed P-value of 0.05 was considered statistically significant. In addition, to examine the association between ECG and all-cause mortality, survival curves were computed using Kaplan-Meier estimates.

This study complies with all relevant ethical regulations and was approved by the Research Ethics Committee of the Universidade Federal de Minas Gerais, protocol 49368496317.7.0000.5149. Since this is a study with secondary data, obtained for clinical reasons, a waiver of informed consent was required and granted by the Research Ethics Committee. Researchers signed terms of confidentiality and data utilization.

## Results

From a dataset of 1,773,689 patients, 1,558,421 primary care patients over 16 years old underwent a valid ECG recording during 2010 to 2017 and, therefore, were included (mean age 51.6 [SD17.6] years, 4.8% were ≥80 years old, and 40.2% were male). Hypertension was reported in 31.6% of patients, diabetes in 6.5%, dyslipidemia in 3.9%, Chagas disease in 2.2%, previous myocardial infarction in 0.7%, COPD in 0.7%, and 7.0% reported current smoking.

The prevalence of AF overall was 1.33% (n = 20,782) with higher prevalence with advancing age, particularly from 70 years, reaching 7.0 % in octogenarians (Table 1). AF patients were older than non AF patients with a mean age of 71.1 (SD: 13.7) years compared to 51.6 (SD 17.6) years, respectively. Men had a higher prevalence than women. All comorbidities were significantly more prevalent in AF patients, adjusted for age and sex, except for diabetes and smoking (Table 2).

**Table 1:** Prevalence of atrial fibrillation according to age distribution and sex.

Age (years)	N		Prevalence n; (%)	

	Male	Female	Male	Female

17–30	89,999	126,966	150 (0.17)	87 (0.07)
31–40	82,116	140,047	280 (0.34)	206 (0.15)
41–50	106,986	181,680	554 (0.52)	405 (0.22)
51–60	128,794	192,175	1,344 (1.04)	954 (0.50)
61–70	113,793	150,517	2,690 (2.36)	1,978 (1.31)
71–80	74,344	96,774	3,698 (4.97)	3,257 (3.37)
>80	31,010	43,220	2,610 (8.42)	2,569 (5.94)
Total	627,042	931,379	11,326 (1.81)	9,456 (1.02)

**Table 2:** Baseline data by prevalence of atrial fibrillation.

Variable	Patients with AF (n = 20,782)	Patients without AF (n = 1,445,584)	Age- and Sex-adjusted OR (95% CI)	Multivariable-adjusted* OR (95% CI)

Age (years)	71.1 ± 13.7	51.6 ± 17.6		1.08 (1.08–1.08)
Male sex	11,326 (54.5)	615,716 (40.0)		1.79 (1.74–1.84)
Current smoking	1,368 (6.6)	107,447 (7.0)	0.94 (0.89–0.99)	0.94 (0.89–0.99)
Hypertension	10,288 (49.5)	482,353 (31.4)	2.14 (2.09–2.20)	1.31 (1.27–1.34)
Diabetes	1,769 (8.5)	99,701 (6.5)	1.34 (1.28–1.41)	0.99 (0.95–1.05)
Dyslipidemia	1,115 (5.4)	59,475 (3.9)	1.41 (1.33–1.50)	1.09 (1.03–1.16)
Chagas disease	1,479 (7.1)	33,111 (2.2)	3.48 (3.30–3.67)	3.08 (2.91–3.25)
MI	377 (1.8)	11,227 (0.7)	2.51 (2.26–2.79)	1.74 (1.56–1.93)
COPD	338 (1.6)	10,928 (0.7)	2.31 (2.07–2.58)	1.48 (1.33–1.66)

Data are presented as mean (±SD) or number (%). OR, odds ratio; CI, confidence interval; COPD, chronic obstructive pulmonary disease; MI, myocardial infarction *multivariable models adjusted for variables in the table.

**Figure 2:**
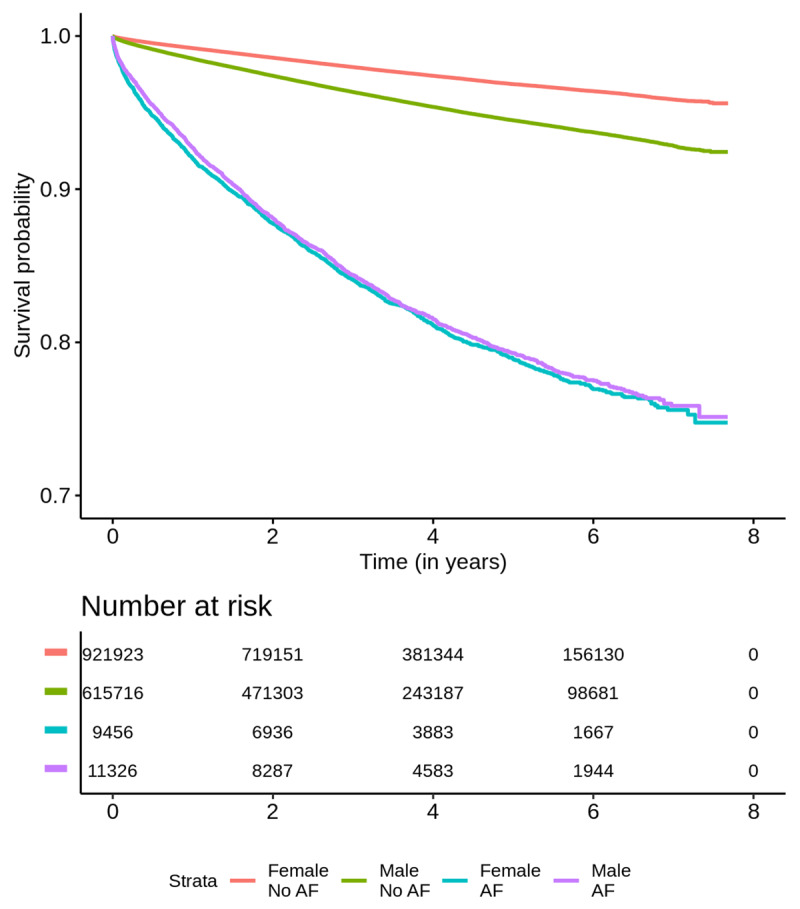
Kaplan-Meier survival curves for atrial fibrillation according to sex.

The overall mortality rate was 3.34% over a mean follow-up time of 3.68 years (Table 3). In univariate analysis, AF was strongly associated with death from all causes (HR 5.85, 95% CI 5.65–6.04). After adjustment for age, sex and clinical conditions, AF had a 2.10 fold risk of all-cause mortality (HR 2.10, 95% CI 2.03–2.17). In multivariable analysis by sex, adjusted for age and comorbidities, women with AF had a higher risk of death for all causes (HR 2.59, 95% CI 2.47–2.73) than men with AF (HR 1.83, 95% CI 1.74–1.91) (Figure 2).

**Table 3:** Event rates in patients with atrial fibrillation.

Outcome	N	Events	%	Event rate (per 100 person-years)	95%CI

All-cause death	20,782	3,701	17.81	1.78	(1.72–1.84)
Cardiovascular death	20,782	398	1.91	1.89	(1.73–2.13)

For the secondary endpoint, cardiovascular mortality, AF was also a predictor of risk after adjustment for age, sex and clinical conditions (HR 2.06, 95% CI 1.86–2.29). Female patients also had an increased risk for cardiovascular mortality (HR 2.62; 95% CI 2.24–3.06) compared to men (HR 1.71; 95% CI 1.62–1.80); p < 0.001 (Table 4).

**Table 4:** Adjusted Hazards of Atrial Fibrillation for Mortality.

Type of event	HR (CI 95%)			p-value

	All patients^+^	Male*	Female*	

Death for all causes	2.10 (2.03–2.17)	1.83 (1.74–1.91)	2.59 (2.47–2.73)	<0.001
Cardiovascular death	2.06 (1.86–2.29)	1.71 (1.62–1.80)	2.62 (2.24–3.06)	<0.001

HR, hazard ratio; CI, confidence interval. + adjusted for age, sex and comorbidities. * adjusted for age and comorbidities.

## Discussion

### Prevalence

In a dataset of 1,558,421 Brazilian patients, the prevalence of AF was 1.33%. AF was a risk factor for all-cause and cardiovascular mortality in models adjusting for comorbidities. Female patients with AF had a higher risk of overall and cardiovascular mortality compared with males.

We found the overall prevalence of AF to be lower than in a previous study (1.33 vs. 1.8%) made in the same population [4], probably due to a larger cohort studied in a different period of time with another methodology for AF diagnosis and the exclusion of atrial flutter in the present study. Besides that, AF can be paroxysmal and as we only included the first ECG from each patient, AF might have been diagnosed in the second or third ECG.

A Dutch ECG database of primary care patients with equivalent clinical conditions reported a similar prevalence [19]. On the other hand, a New Zealand primary care population had a higher prevalence (2.8%) [20], using a different age range (35 to 74 years old), which could have influenced the results, since AF prevalence is highly dependent on age.

Male sex had a higher prevalence of AF in our study. The higher prevalence of AF in men has already been reported in several studies in high and low- and middle income countries (LMIC) [1]. Our rates of associated clinical conditions were lower compared with other populations [21,22], probably because the comorbidities were self-reported. As reported in our previous study. Chagas disease, previous myocardial infarction, COPD and hypertension are the clinical conditions more strongly associated with AF prevalence.

### Mortality from all causes

AF is a well-known risk factor for death from all causes and cardiovascular deaths [5], in part, because of the association with heart failure and cerebrovascular diseases [23]. Current guidelines for AF treatment recommend thromboembolic risk assessment with appropriate anticoagulation if indicated, rhythm control in symptomatic patients and appropriate treatment of cardiac risk factors [8]. Anticoagulation treatment of AF can decrease overall mortality in AF patients [24]. In Brazil, the majority of AF patients are treated by primary care physicians with low anticoagulation compliance. In our previous study, we found that only 1.5% of primary care patients reported the use of warfarin [4]. Even in a Brazilian tertiary center, only 55% of AF patients at high risk were receiving dose-adjusted warfarin [25]. The use of new anticoagulant drugs is low in public health care as they are expensive and not provided by the Brazilian government.

Previous studies had reported higher mortality in women with AF than men. In a systematic review and meta-analysis of thirty cohort studies that included more than four million participants, AF was associated with a higher risk of all-cause mortality, stroke, cardiovascular mortality, cardiac events and heart failure in women compared to men [26]. AF in women was associated with a 12% greater risk of all-cause mortality than in men [27].

It is unclear what factors predispose to a higher risk of mortality in women versus men. Differences in hormones and in electrical and structural characteristics might help explain variation in incidence, prevalence, burden, and complications associated with AF in women and men [27]. Physiological or psychosocial differences between women and men could have some impact in cardiovascular risk [26]. Therapy with anticoagulants may have a distinct response in women, with a consequent higher risk of bleeding [26]. Other possibility is that women are undertreated with anticoagulation therapy [28]. However, contemporary global data showed no difference in anticoagulant use in men and women with nonvalvular AF [29]. Future research is needed to better evaluate these possible mechanisms.

### Cardiovascular mortality

Cardiovascular mortality was also higher in AF patients. The association between AF and higher risk of stroke and heart failure may underlie this finding [2]. The GARFIELD AF registry reported 1.46/100 persons-year rate of cardiac deaths with heart failure as the leading cause. At two-year follow-up, all-cause death was the most frequent primary outcome in this population with similar rates for cardiovascular and non- cardiovascular mortality [30].

### Risk assessment and anticoagulation treatment

Our study reported a higher risk of mortality in AF patients. This finding has major implications in public health policy, particularly in LMIC, as Brazil, in where there are no specific protocols for AF screening and treatment in the primary public health system. Anticoagulation management in primary care also remains a challenge, since primary health care personnel are not trained in the anticoagulation control and direct oral anticoagulants are not available in most LMIC. Hence, stroke is the second leading cause of death worldwide with a significant impact on the economy [8,31]. Treatment strategies for AF with better anticoagulation compliance and cardiologic evaluation should be reassessed. Financial support allocation for primary care should be reinforced.

The automated reports may very occasionally differ in reporting AF. The Minnesota Code does not state any criteria for AF but simply assigns code 8-3-1 to this arrhythmia. However, if the Glasgow program reports for example ‘Probable atrial fibrillation with complete AV block’ this has been automatically assigned only a code 6-1-0, i.e. Complete (third degree) AV block. Thus, the strategy of using a report of AF in either the diagnostic interpretation or the Minnesota Code was adopted.

## Limitations

As AF is often asymptomatic, can be paroxysmal, and because we used the first available ECG in the data set, the prevalence of AF may be underestimated. Comorbidities and medication data were self-reported, and thus might have been underreported and misclassified non-randomly. We did not have data from heart failure in our patients; therefore, it was not included in our statistical model. For the cross-sectional clinical correlates of AF we cannot establish temporality and note that risk factors may have a bidirectional relationship with AF (e.g. myocardial infarction). For both the clinical correlates and the mortality of AF, we acknowledge the observational nature of the data; we cannot rule out residual confounding and we cannot establish causal relations. Lazy Associative Classifier used for ECG report classification has good accuracy, sensitivity, and positive predictive value, but it is not free from errors. The information from the national mortality system is heterogeneous among the regions of Minas Gerais and misclassification in the basic cause of death can occur. The probabilistic linkage also has some issues such as a less than perfect sensitivity and the possibility of false pairing. To minimize this problem, we defined a high cut off point for true pairs and made a manual revision in the doubtful cases.

## Further research

Our study was innovative because it was the first to assess mortality in AF in a large Brazilian primary care population. However, further studies with reliable clinical and hospitalization data are needed to better understand epidemiological features of AF in our population. As the study involves a large electronic cohort with millions of patients, we believe that these small imperfections do not impact on our findings.

## Conclusions

AF is an independent risk factor for all-cause and cardiovascular mortality in patients of Brazilian primary care centers, with increased risk in women for overall and cardiovascular mortality. Public health policy should improve surveillance in this population with better prevention and treatment strategies, as evidence-based treatment for cardiac conditions (heart failure, hypertension and coronary artery disease), guidelines for management of oral anticoagulation and education of primary health care professionals.
